# Ultraviolet Light Treatment of Titanium Enhances Attachment, Adhesion, and Retention of Human Oral Epithelial Cells via Decarbonization

**DOI:** 10.3390/ma14010151

**Published:** 2020-12-31

**Authors:** Kourosh Nakhaei, Manabu Ishijima, Takayuki Ikeda, Amirreza Ghassemi, Juri Saruta, Takahiro Ogawa

**Affiliations:** 1 Weintraub Center for Reconstructive Biotechnology, Division of Advanced Prosthodontics, UCLA School of Dentistry, Los Angeles, CA 90095, USA; kourosh1988@ucla.edu (K.N.); manab612@gmail.com (M.I.); ikeda.takayuki@nihon-u.ac.jp (T.I.); a.r_ghassemi_dent@yahoo.com (A.G.); togawa@dentistry.ucla.edu (T.O.); 2 Department of Complete Denture Prosthodontics, Nihon University School of Dentistry, 1-8-13 Kanda Surugadai, Chiyoda-ku 101-8310, Tokyo, Japan; 3 Section of Periodontics, Department of Applied Dental Medicine, Southern Illinois University School of Dental Medicine, 2800 College Ave, Alton, IL 62002, USA; 4 Department of Oral Science, Graduate School of Dentistry, Kanagawa Dental University, 82 Inaoka, Yokosuka 238-8580, Kanagawa, Japan

**Keywords:** soft tissue, hydrocarbon, photofunctionalization, dental and orthopedic implants

## Abstract

Early establishment of soft-tissue adhesion and seal at the transmucosal and transcutaneous surface of implants is crucial to prevent infection and ensure the long-term stability and function of implants. Herein, we tested the hypothesis that treatment of titanium with ultraviolet (UV) light would enhance its interaction with epithelial cells. X-ray spectroscopy showed that UV treatment significantly reduced the atomic percentage of surface carbon on titanium from 46.1% to 28.6%. Peak fitting analysis revealed that, among the known adventitious carbon contaminants, C–C and C=O groups were significantly reduced after UV treatment, while other groups were increased or unchanged in percentage. UV-treated titanium attracted higher numbers of human epithelial cells than untreated titanium and allowed more rapid cell spread. Hemi-desmosome-related molecules, integrin β4 and laminin-5, were upregulated at the gene and protein levels in the cells on UV-treated surfaces. The result of the detachment test revealed twice as many cells remaining adherent on UV-treated than untreated titanium. The enhanced cellular affinity of UV-treated titanium was equivalent to laminin-5 coating of titanium. These data indicated that UV treatment of titanium enhanced the attachment, adhesion, and retention of human epithelial cells associated with disproportional removal of adventitious carbon contamination, providing a new strategy to improve soft-tissue integration with implant devices.

## 1. Introduction

Oral or orthopedic implants that are transcutaneous or transmucosal, such as most dental implants, maxillofacial epithesis anchors, and various connection devices in orthopedic reconstruction, are prone to bacterial contamination by resident and harbored flora on skin and mucosal surfaces, leading, in some cases, to soft-tissue infections and failure of the implant treatment [1]. Dental implants are the most challenging in this regard, due to the hostile and diverse microbial environment of an oral cavity, which could contain more than 600 different bacterial species and be subject to infectious disease such as peri-implantitis [2,3]. A systematic review paper based on 1497 participants and 6283 implants reported peri-implantitis in 18.8% of participants and 9.6% of implants [4,5]. Thus, control of bacterial infection is one of the most important factors for successful implant treatments [6,7].

Establishing an early soft-tissue attachment or seal to the surface of implant devices reduces the chance of bacterial invasion at the implant-transmucosal or implant-transcutaneous junction, and thus increases the chance of long-term treatment success [8,9]. Previous studies sought to better understand the mechanisms by which soft tissue attaches to and forms a seal around implants. For instance, immunoelectron microscopy revealed that epithelial cells responsible for attaching soft tissue to the implant surface act via the internal basal lamina and hemidesmosomes at the plasma membrane and the receptor to ligand binding of integrin to laminin [10,11,12,13]. However, despite the qualitative and observational studies examining successfully established implant-soft tissue interface, a methodology for enhancing the probability, speed, and strength of the soft-tissue attachment and seal has not been established.

The surface treatment of titanium with ultraviolet (UV) light alters its physicochemical and biological properties. UV treatment of titanium enhanced the proliferation and differentiation of osteoblasts, as compared to untreated titanium [14,15]. UV treatment substantially increased the bone volume formed around titanium and thereby the strength of bone-implant integration [16,17,18]. Clinical studies reported a better clinical success and shorter healing time required for bone integration for UV-treated titanium implants [19,20,21,22,23]. The effect of UV treatment on the epithelial cells and soft tissue is unknown. We hypothesized that surface treatment of titanium implants with UV light enhances the attachment, spreading, adhesion, and retention of epithelial cells to the implant surfaces, and tested that hypothesis herein using human oral epithelial cells in vitro.

## 2. Materials and Methods

### 2.1. Titanium Disks and UV Treatment

Grade 2 commercially pure titanium disks (20 mm in diameter and 1.5 mm in thickness) with machined surfaces were prepared by autoclaving followed by storage under dark ambient conditions for 4 weeks to standardize the titanium properties, based on the documented time-dependent degradation of titanium on biological capabilities [24,25,26]. UV treatment was performed using a photo device (TheraBeam Super Osseo, Ushio Inc., Tokyo, Japan) for 12 min, with the chemical composition of the surfaces determined by X-ray photoelectron spectroscopy (XPS) (Axis Ultra DLD spectrometer, Kratos Analytical, Shimadzu, Kyoto, Japan) and the hydrophilicity evaluated by measuring the contact angle of 10 µL ddH_2_O.

### 2.2. Human Oral Epithelial Cells Culture

Human oral epithelial cells (Celprogen Inc., Torrance, CA, USA) were used for this experiment. Cells were cultured in culture dishes (Falcon, Glendale, AZ, USA) with commercial human oral epithelial cell medium containing serum (Celprogen Inc.). At 80% confluency, cells were detached by using 0.05% Trypsin-EDTA (Gibco, Life Technologies, Grand Island, NY, USA) and subdivided, with the culture medium changed every three days, and cells between passage six and eight used for all experiments. Cells were seeded onto disks placed in 12-well culture plates (Fisher Scientific, Pittsburgh, PA, USA), at a density of 3 × 10^4^ cells/well, and incubated at 37 °C with 5% CO_2_. For cell morphology and morphometry, a cell density of 1 × 10^4^ cells/well was used for seeding.

### 2.3. Initial Cell Attachment Assay

The number of cells initially attached to each surface was colorimetrically quantified after 3 and 24 h incubations. Briefly, the cells on the disks were rinsed with phosphate-buffered saline (PBS) twice and then moved to new 12-well culture plates, to remove floating cells. Then, 100 μL of WST-1 reagent (Roche Applied Science, Indianapolis, IN, USA) was added to the cultures, at 37 °C, for 1 h, and then the absorbance was measured in each well, at a wavelength of 450 nm, using a plate reader (Biorad, Hercules, CA, USA). For the staining, cells were fixed in 10% formalin for 8 min and then stained with the fluorescent dye rhodamine phalloidin (Invitrogen, Grand Island, NY, USA) and observed by using fluorescence microscopy to confirm the result of the colorimetric assay. The phalloidin specifically binds F-actin and fluoresces red-orange. The specimens were embedded in mounting medium (Vectashield, Fisher Scientific, Pittsburgh, PA, USA) and observed by using a confocal laser-scanning microscope (CLSM) (Leica TCS-SP5 STED confocal-multiphoton microscope, Leica Microsystems, Heidelberg, Germany). The coverage area of epithelial cells was measured by using an ImageJ (NIH, Bethesda, ML, USA).

### 2.4. Cytomorphology and Cytomorphometry

CLSM was used to examine cell morphology and cytoskeletal arrangement of epithelial cells on the titanium surfaces after 3 and 24 h of culture and after staining with rhodamine phalloidin, as above. Cell area, perimeter, and Feret’s diameter were quantified by using an ImageJ (NIH).

### 2.5. Adhesion Protein Assay

The cells were stained with mouse anti-human integrin β4 monoclonal antibody (Abcam, Cambridge, MA, USA), followed by a fluorescein isothiocyanate (FITC)-conjugated anti-mouse secondary antibody (Abcam). The expression of integrin β4 was observed, using the CLSM, and quantified by ImageJ (NIH).

### 2.6. Real-Time Quantitative Polymerase Chain Reaction (qPCR)

Total cellular RNA was extracted, using TRIzol reagent (Invitrogen, Carlsbad, CA, USA) and Direct-zol RNA MiniPrep kit (Zymo Research, Irvine, CA, USA) after 12 h of culture. Quantitative PCR was performed by using QuantStudio 3 System (Thermo Fisher Scientific, Canoga Park, CA, USA) in a 20 μL volume. The mRNA expression of the target genes integrin β4 (Hs00173995_m1) and laminin-5 (Hs00165042_m1) were determined. GAPDH (Hs02758991_g1) was used as the endogenous control.

### 2.7. Cell Detachment Assay

To evaluate a substantial cell retention force to titanium surfaces, we performed cell detachment assays. After 24 h of incubation, cultures were rinsed with PBS twice and then moved to new culture plates. Half of the disks were agitated (amplitude 10 mm; frequency 30 Hz) in 0.025% Trypsin solution, for 10 min, to detach cells from the surface, while the other half were statically incubated for 10 min. The numbers of cells were colorimetrically measured, using WST-1 reagent. Cell retention rate was calculated as [(remaining cell on the disks after detachment)/(remaining cell on the statically cultured disks)] × 100 (%). Cells were observed by using a scanning electron microscope (SEM; Nova 230 NanoSEM; FEI, Hillsboro, OR, USA) before and after detachment, as previously described [27].

### 2.8. Laminin Coating of Titanium Disks

Untreated titanium disks were immersed in 500 μL of Tris buffer solution containing 100 μg of human laminin-5 (Kerafast, Boston, MA, USA) and incubated for 2 h at 37 °C. After the coating of laminin-5, disks were twice washed gently with PBS, to remove any unattached proteins. Cells were seeded immediately after the coating, and the cells attaching to the surface were colorimetrically measured after 3 and 24 h of incubation.

### 2.9. Statistical Analysis

Three samples were analyzed for all cell culture experiments, except for the cell morphometry (six samples), and all experiments were repeated three times. The Mann-Whitney U test was used to examine the difference between the untreated and UV-treated groups at each time point, except for the evaluation of the effect of laminin coating, with *P*-values less than 0.05 considered significant. One-way ANOVA with Bonferroni multiple comparisons was used to identify differences between groups for the laminin coating, with *P*-values less than 0.05 considered significant.

## 3. Results

### 3.1. UV Treatment Induced Decarbonized and Super-Hydrophilic Surfaces

**Figure 1:**
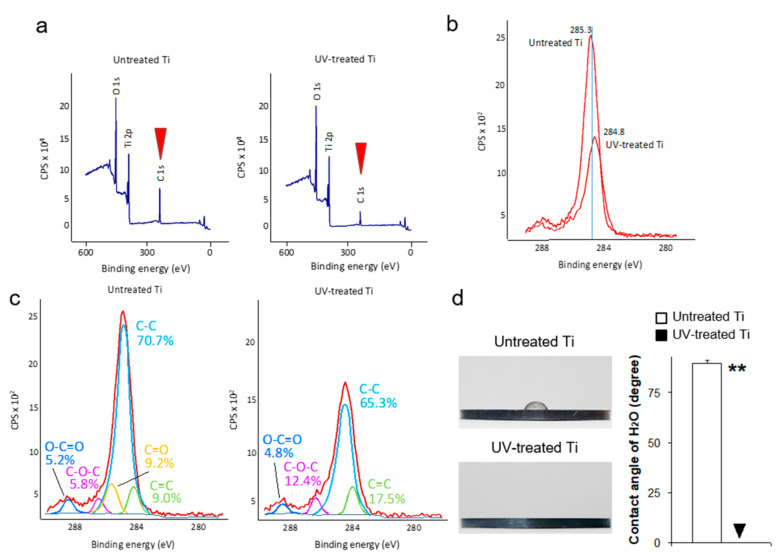
Ultraviolet (UV)-light-induced physicochemical changes on the machined titanium surfaces. (**a**) X-ray photoelectron spectroscopy (XPS) spectrum for the untreated control and UV-treated titanium surfaces. The red arrowhead represents the C1s peak. (**b**) Spectrum showing the C1s peak for the untreated control titanium and UV-treated titanium. (**c**) Spectrum representing the peak fitting analysis for C1s. (**d**) A total of 10 µL of H_2_O was placed on the untreated and UV-treated titanium, to evaluate surface hydrophilicity. The line graph shows the average contact angle of H_2_O placed on each surface (** *P* < 0.01).

First, we analyzed UV-induced physicochemical changes to the titanium surfaces. UV treatment substantially reduced the amount of surface carbon on the titanium disks by approximately 40%, with average atomic percentages of carbon on the untreated and UV-treated surfaces of 46.1 ± 0.9% and 28.6 ± 0.5%, respectively (Figure 1a). The value of binding energy for C1s was 285.3 and 284.8 for the untreated titanium and UV-treated titanium, respectively, with the spectrum moved slightly to the right side of the figure after UV treatment (Figure 1b). Further peak fitting analysis for the carbon element revealed the spectrum of C1s broken down into several groups. The untreated titanium C1s spectrum was composed of O–C=O, C–O–C, C=O, C–C, and C=C. The UV treatment disproportionally altered this spectrum, removing C=O and increasing the proportion of C-O–C and C=C on the surface (Figure 1c). The decarbonized titanium surfaces represented a super-hydrophilic property (Figure 1d).

### 3.2. Enhanced Initial Cell Attachment to UV-Treated Surfaces

**Figure 2:**
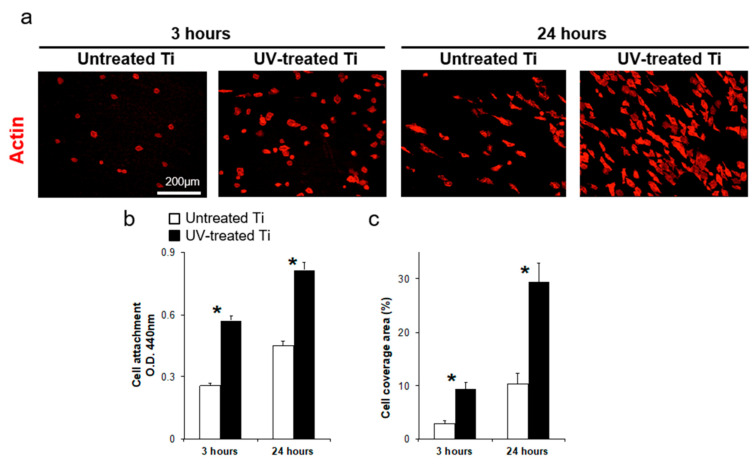
Initial cell attachment of human oral epithelial cells cultured on untreated control and UV-treated titanium surfaces. (**a**) Cells cultured on the untreated control and UV-treated titanium surfaces were stained for actin with rhodamine-phalloidin (red) and observed by using fluorescence microscope after 3 and 24 h of culture. Scale bar = 200 µm. (**b**) Cell number was quantified with a colorimetric-based WST-1 assay (* *P* < 0.05). (**c**) Cell coverage area as a percentage on the untreated control and UV-treated titanium surfaces (* *P* < 0.05).

The confocal fluorescent imaging showed significantly higher numbers of cells on the UV-treated surfaces, with 2.2 and 1.8 times more cells attaching to the UV-treated surfaces after 3 and 24 h of incubation, respectively, compared to numbers on the untreated control surfaces (Figure 2a,b). These numbers indicated an accelerated initial cell attachment to the treated titanium (Figure 2b). Furthermore, the coverage area of epithelial cells attaching to the UV-treated surfaces was 3.2 and 2.9 times larger than that on untreated surface after 3 and 24 h of incubation, respectively (Figure 2c).

### 3.3. Accelerated Cell Spreading on UV-Treated Titanium

**Figure 3:**
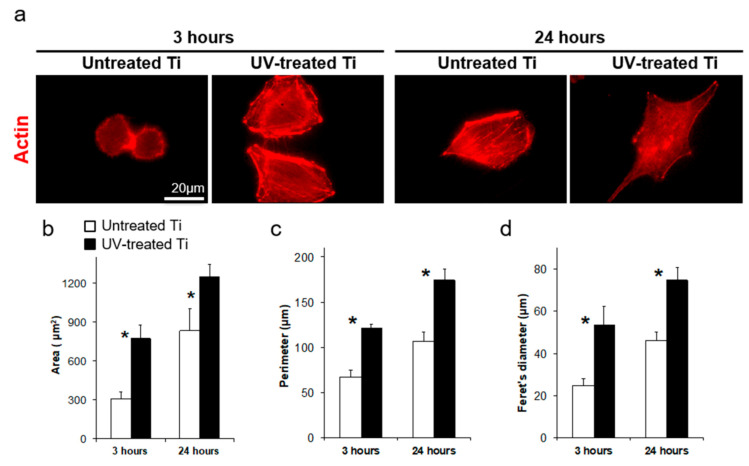
Cytomorphometry of osteoblasts on the titanium surfaces. (**a**) Representative images of initial cell spreading and cytoskeletal development during culturing on the untreated control and UV-treated titanium surfaces after 3 and 24 h of culture. The cell actin was stained with rhodamine phalloidin (red). Scale bar = 20 µm. (**b**–**d**) Bar graphs show the cell morphometric evaluation for cell area, perimeter, and Feret’s diameter (maximum diameter) (* *P* < 0.05).

At 3 h of incubation, cells on the UV-treated surfaces showed spreading with extended filopodia and lamellipodia, while cells on the untreated surfaces remained spherical at the same incubation point (Figure 3a). The cells on the UV-treated surfaces also spread wider in total surface area than those on the untreated surfaces by 24 h of incubation (Figure 3a). Morphometric parameters of cell area, perimeter, and Feret’s diameter on the UV-treated surfaces showed greater values than those measured in the untreated surfaces (Figure 3b–d).

### 3.4. UV Treatment Enhanced the Expression of Adhesion Proteins

**Figure 4:**
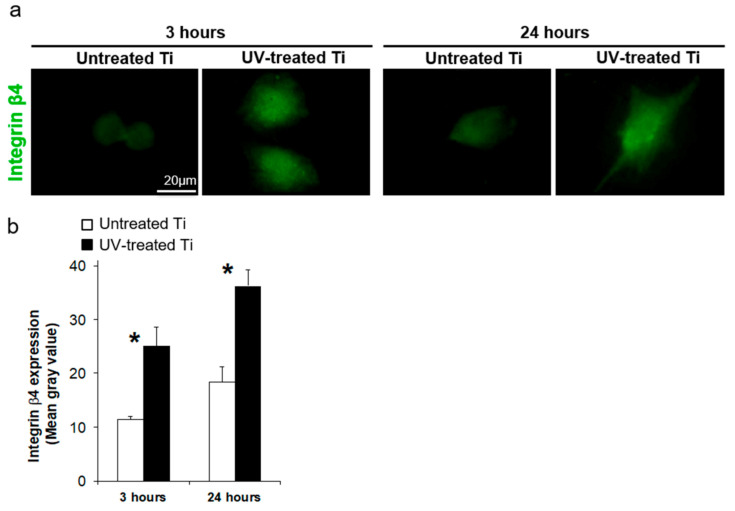
Protein expression of hemidesmosome-related protein, integrin β4. (**a**) Representative images of integrin β4 expression in cells on the untreated control and UV-treated titanium surfaces after 3 and 24 h of culture. Scale bar = 20 µm. (**b**) The bar graph shows image-based quantification of the integrin β4 expression (* *P* < 0.05).

**Figure 5:**
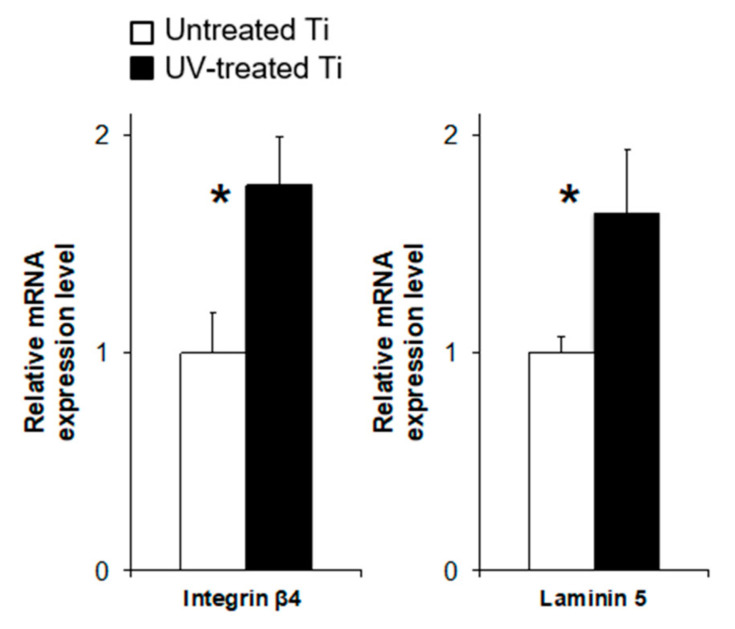
The mRNA expression of hemidesmosome-related proteins, integrin β4 and laminin-5, after 12 h of culture. Relative mRNA expression levels were determined by using the delta–delta CT method with GAPDH as an internal control (* *P* < 0.05).

Expression of integrin β4, a component of hemidesmosomes, was detected in the spreading cells on both titanium surfaces (Figure 4a); however, image analysis revealed 2.2 and 2.0 times higher expression of integrin β4 in cells on the UV-treated titanium than that on untreated titanium after 3 and 24 h of culture, respectively (Figure 4b). The expression levels of integrin β4 and laminin-5 on UV-treated titanium were also 1.8 and 1.6 times higher at the gene level than on untreated titanium at 3 and 24 h of incubation, respectively (Figure 5).

### 3.5. UV Treatment Enhanced Cell Retention

**Figure 6:**
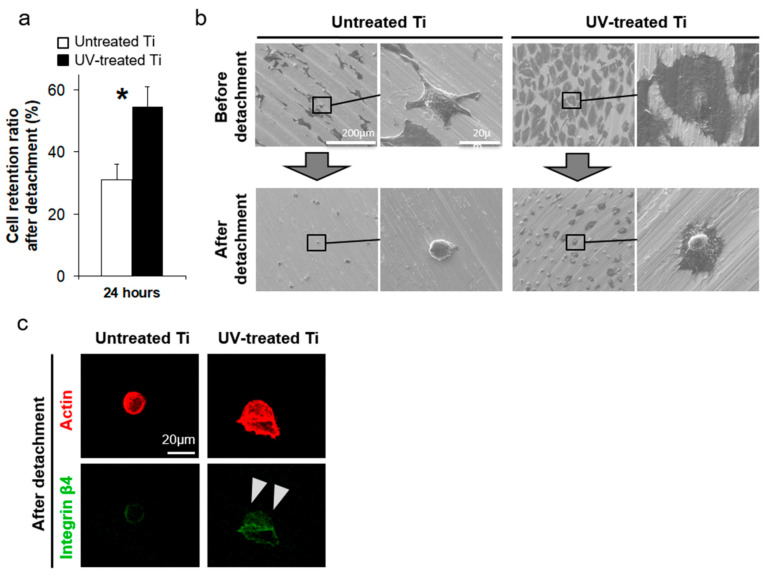
Cell detachment assay for the evaluation of cellular retention force to a titanium surface. After 24 h of culture, cells were chemically and physically detached by agitation in 0.025% Trypsin solution. (**a**) The cell retention ratio was calculated by quantifying the number of cells attached to the titanium surfaces before and after attachment (* *P* < 0.05). Cell retention ratio (%) = (the number of cells attached to the titanium surfaces before detachment)/(the number of cells attached to the titanium surfaces after detachment) × 100. (**b**) Representative scanning electron microscope images of cells before and after detachment on the untreated control and UV-treated titanium surfaces. (**c**) Integrin β4 expression in the cells after detachment (white arrowhead). Scale bar = 20 µm.

Cell retention to the titanium surfaces, measured by using a cell detachment assay, was significantly enhanced by the UV treatment, as compared to the untreated surfaces, with approximately double the number of cells remaining on the UV-treated surfaces, as compared to untreated surfaces after detachment (Figure 6b). The SEM images also showed that the cells remaining on the UV-treated surfaces after cell detachment were much larger than those few cells remaining on the untreated surfaces (Figure 6a). Typical cells on the UV-treated disks still showed lamellipodia, even after detachment (Figure 6a), while cells retained on the untreated disks were spherical, without lamellipodia or filopodia (Figure 6a). The remaining cells on the UV-treated surfaces also showed positive expression of integrin β4, even after detachment (Figure 6c).

### 3.6. Laminin Coating

**Figure 7:**
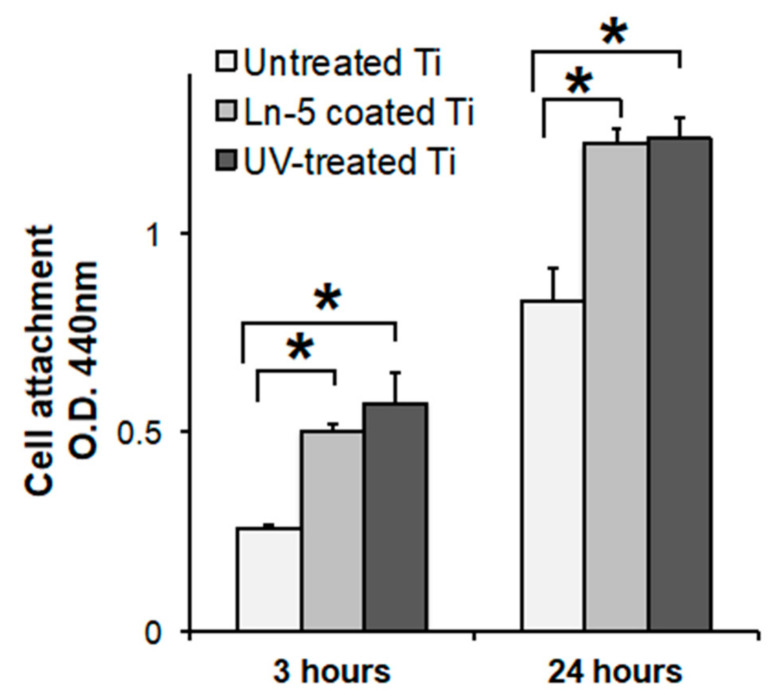
Initial cell attachment of human oral epithelial cells cultured on untreated control, human laminin-5 coated, and UV-treated titanium surfaces. Cell number was quantified with a colorimetric-based WST-1 assay (* *P* < 0.05).

Laminin coating of the titanium disks enhanced cell attachment by 2.2 and 1.5 times compared to that on the untreated controls after 3 and 24 h incubation, respectively, and this enhancement in cell attachment to the titanium surfaces was matched quantitatively by the UV treatment (Figure 7).

## 4. Discussion

XPS analysis showed the existence of carbon on the untreated acid-etched titanium disks and that UV treatment significantly reduced the carbon element from the surfaces (Figure 1b–e), in line with several previous studies of implant surfaces [24,26]. The origin of such carbon elements could be the atmosphere, water and cleaning solutions, and packaging, all of which might make contact with the implant surfaces during manufacturing or shipping. This carbon impurity can affect the hydrophilicity of titanium implant surfaces [28], and thus also cellular behavior [29,30,31,32]. Previous studies examining the effect of carbon contamination to titanium surfaces on bone formation in rats revealed a time-dependent increase in elemental carbon element on titanium surfaces cleaned by acid etching after four weeks in the atmosphere. [24,26]. Interestingly, they also showed less bone formation surrounding four-week-old titanium implants, as compared with newly manufactured implants, using an osteotomy model. This phenomenon was named the “biological aging of titanium”; however, the effect of the biological aging of titanium on soft tissue has not been understood. This study is the first to address the important question of how the natural biological aging of titanium affects soft tissue. To date, researchers have made various attempts to give titanium a new function [33,34]. We have succeeded in adding a new function of strengthening the adhesion of soft tissue cells by using UV light.

In general, UV-light irradiation of titanium surfaces causes two different chemical reactions, namely photolysis and photocatalysis, both of which decompose organic compounds. Photolysis is the direct decomposition of organic compounds caused by high-intensity light. At the same time, the titanium dioxide passive layer photocatalytically decomposes carbon compounds under the treatment with UV light. A previous study has confirmed the titanium dioxide passive layer on the same type of acid-etched titanium used in this present study [17]. The exposure of titanium dioxide to UV light excites electrons to move from the valence band to the conduction band, and thus catalyze the chemical reaction [35]. Thus, the UV treatment, directly and indirectly, decomposes impurities from the titanium surface. The present study addressed the detailed mechanisms of the effect of UV treatment on the decomposition of carbon-including compounds and revealed the specific chemical bonds broken by UV light on the acid-etched titanium surfaces.

The UV treatment conducted in the present study significantly altered the behavior of human oral epithelial cells cultured on titanium surfaces, increasing both the number and spreading of epithelial cells attached to the titanium surfaces. One explanation of the enhanced initial cellular attachment is the electrostatic mechanism occurring with the UV treatment of titanium [17], whereby the UV irradiation induces a photocatalytic chemical reaction on the titanium surface that could increase cell attraction and thus attachment via the UV-induced positive charging of the titanium surfaces attracting the negatively charged proteins and cells [14,15]. In fact, UV-enhanced cellular attachment can be abrogated when positively charged UV-treated titanium surfaces are electrostatically neutralized by either removing the electric charge or masking with monovalent anions [15].

Epithelial cells attach to biomaterial surfaces through the hemidesmosomes of adhesion plaques [10,36], and enhancing this interaction is the key to soft-tissue integration on biomaterial surfaces. Herein we showed that the key hemidesmosome-associated proteins, integrin β4 and laminin-5, were upregulated at the gene level on our UV-treated surfaces, and such enhanced expression would likely boost the retention ability of epithelial cells for the UV-treated titanium surfaces, as compared to untreated surfaces, as represented by the increased numbers of cells attached against enzymatic and mechanical detachments (Figure 5). An enhancement of cell spreading, as observed herein, could further affect the mechanisms of cellular adhesion and retention. The cellular spreading of anchorage-dependent cells, such as the epithelial cells used in this study, also significantly affects subsequent proliferation and function of cells [37,38]. Based on these data together, further studies are needed to understand the detailed mechanisms underlying the UV-treatment-induced enhancement of hemidesmosome function.

Previous studies reported techniques aimed at enhancing epithelial attachment to titanium surfaces, including physicochemical alterations and coating [36,39,40,41]. For example, the hydrothermal treatment of a titanium surface with calcium or magnesium increased epithelial and fibroblastic cell adhesion [41], while other studies reported that surface coating with insulin-like growth factor-1 or laminin-5 significantly enhanced epithelial attachment to titanium surfaces [36,39,40]. The advantages and disadvantages for each such technique must be considered for each biomaterial and biological setting. The use of bioactive proteins, such as a cytokine or growth factor, should be well managed, to maintain its activity, while coating techniques have the common problem that the interface between base materials and coating materials is prone to detachment. From this point of view, UV treatment could be an easy and simple choice. First, UV treatment does not affect the surface morphology, and this is an important point for modern implants that often have a unique surface morphology to enact specific biological effects, such as a micro-rough surface designed to enhance osteoblastic differentiation [42]. Further, the combination of a morphologically optimized surface and the UV treatment could synergistically enhance soft-tissue sealing, as indicated with the testing of osteoblasts with different types of surfaces with different surface morphology, including smooth, acid-etched, and nano-featured surfaces [16,43,44]. The second advantage of UV-treated titanium surfaces is its antimicrobial property. Other studies reported that UV treatment of titanium surfaces significantly reduced bacterial attachment and biofilm formation for different strains of bacteria, including *Staphylococcus aureus* and *Streptococcus pyogenes* [45,46]. Bacterial adhesion on titanium implant surfaces has a strong influence on healing and long-term outcome of dental implants [47,48]. Further in vivo and clinical studies focusing on the soft tissue surrounding implants are now warranted, to gain a deeper understanding of how UV treatment might affect titanium implant surfaces and treatment success.

## 5. Conclusions

The UV treatment halved the atomic percentage of surface carbon and converted hydrophobic surfaces to superhydrophilic surfaces. The results of in vitro experiments using human oral epithelial cells revealed that the UV-treated titanium surfaces showed increased initial cell attachment, as compared to untreated controls, and these cells spread faster and more broadly than the cells on the untreated titanium. The UV-treated surfaces also had increased cellular retention that was directly associated with the increased expression of hemidesmosome-related proteins both at gene and protein levels. These data indicated that UV treatment of titanium at the transmucosal and transcutaneous part of implants could improve soft tissue integration and provide a new strategy for preventing exogenous infection and securing long-term stability and function of implants.

## Data Availability

The data presented in this study are available on request from the corresponding author.
